# Aggressive desert goby males also court more, independent of the physiological demands of salinity

**DOI:** 10.1038/s41598-018-27651-3

**Published:** 2018-06-19

**Authors:** Topi K. Lehtonen, P. Andreas Svensson, Bob B. M. Wong

**Affiliations:** 1 School of Biological Sciences, Monash University, 3800 Victoria, Australia; 2 Department of Biology and Environmental Science, Linnaeus University, 39231 Kalmar, Sweden

## Abstract

Both between- and within-individual variation in behaviour can be important in determining mating opportunities and reproductive outcomes. Such behavioural variability can be induced by environmental conditions, especially if individuals vary in their tolerance levels or resource allocation patterns. We tested the effects of exposure to different salinity levels on male investment into two important components of mating success–intrasexual aggression and intersexual courtship–in a fish with a resource defence mating system, the desert goby, *Chlamydogobius eremius*. We found that males that were more aggressive to rivals also exhibited higher rates of courtship displays towards females. Contrary to predictions, this positive relationship, and the consistency of the two behaviours, were not affected by the salinity treatment, despite the physiological costs that high salinity imposes on the species. Moreover, over the entire data-set, there was only a marginally non-significant tendency for males to show higher levels of aggression and courtship in low, than high, salinity. The positive correlation between male aggression and courtship, independent of the physiological demands of the environment, suggests that males are not inclined to make contrasting resource investments into these two key reproductive behaviours. Instead, in this relatively euryhaline freshwater species, typical investment into current reproductive behaviours can occur under a range of different salinity conditions.

## Introduction

It is well established that behavioural differences among individuals can influence mating opportunities and reproductive success, while within-individual variation can also be important^1–4^. Such within-individual behavioural variation may arise, for instance, due to allocation trade-offs, with limited resources shared between different components of current reproductive effort, or current reproduction traded against future reproduction and survival^5^. For example, investment by males into sexual advertisement may come at a cost to parental effort, as seen in collared flycatchers, *Ficedula albicollis*, in which experimental manipulation of the cost of a male sexual ornament resulted in changes to male parental investment^6^. In many species, individuals also need to invest both in intrasexual competition (such as a territorial aggression) and sexual signalling (such as courtship displays)^7–9^. This potential trade-off is particularly interesting because both aggressive and sexual displays can inform rivals and potential mates about the quality or motivation of the signaller and, in so doing, affect reproductive success^10,11^. Traditionally, indicator models^12,13^ and empirical studies^11^ have suggested that traits involved in male-male competition and mate attraction should be positively correlated. However, other studies suggest that this is not necessarily the case^14,15^. For example, in *Acheta* and *Gryllus* crickets, sexual signalling effort was not positively correlated with aggression, even though both acoustic signalling and aggressive interactions are important components of mating success in these species^16,17^. Thus, investment in intrasexual competition and mate attraction may not always be positively associated.

Both between- and within-individual variation in reproductive behaviours are likely to be strongly influenced by environmental conditions. Since the environment can impact the amount of available resources, patterns of resource allocation can be influenced by challenging environmental conditions^18–20^. Selection may also favour variability among individuals in how they respond to environmental factors^21–24^, and environmental conditions can affect the relationship between different behavioural traits. For example, in three-spined sticklebacks, *Gasterosteus aculeatus*, exposure to predatory fish resulted in a more pronounced behavioural correlation between boldness and aggressiveness, with the relationship arising from both selective predation and behavioural plasticity^25^.

Salinity and salinity fluctuations have been identified to be among the most important factors that influence species distributions and community structure in aquatic animals^26–29^. Salinity can also affect key reproductive behaviours, both between and within species. In this respect, osmotic adjustments to changing salinity levels and key reproductive behaviours (such as aggression and courtship) share important hormonal controls, especially neurohypophysial hormones and corticosteroids^30–33^. In a gobiid fish, *Pomatoschistus minutus*, short-term manipulations of salinity levels were found to affect male nest-building behaviour differently depending on the size of the nest builder^34^. More generally, for populations and species occupying environments with fluctuating salinities, or a range of more constant salinity levels, salinity provides a particularly relevant context for assessing consistency of reproductive behaviours under different environmental conditions. This is underscored by the fact that higher salinity levels, and adjustment to such conditions, are often associated with increased metabolic demands^27,29,35,36^.

We assessed the effect of salinity conditions on the relationship between male aggression and courtship effort in a fish, the desert goby, *Chlamydogobius eremius*. The desert goby is a small (~5 cm), colourful species native to the springs and rivers of the Lake Eyre Basin in Central Australia^37^, where it commonly encounters varying salinity levels ranging from freshwater conditions to ~100 ppt (authors’ own observations). Exposure to higher salinity levels in desert gobies results in a higher metabolic rate, presumably because of increased metabolic costs of osmoregulation^35^. Nevertheless, male desert gobies may compete for, and establish, nests under rocks and aggressively defend their territories against rivals under a range of different salinity levels^37,38^. Males must also rely on conspicuous and colourful courtship displays (with erect dorsal and anal fins, jerky movements, and movements leading towards the nest) to attract females for mating, with investment in both aggression and courtship being pivotal to male mating success. We previously showed that exposure to different salinities, along with social experiences, can impact certain reproductive behaviours^38^. In particular, males were more aggressive towards rivals after a recent encounter with a female, compared to a recent encounter with another male, under low (but not high) salinity treatment. In contrast, courtship effort of males was unaffected by both salinity and social experience. In that study, however, male aggression and courtship were each investigated in isolation and not recorded repeatedly. Here, by expanding on the study of Lehtonen *et al*.^38^, and capitalising on a larger data-set, we set out to investigate the effect of salinity on the consistency of intrasexual aggression and courtship displays within individuals, the relationship between the two reproductive behaviours, and the overall investment into the two behaviours. We predicted that higher salinity can impede the consistency of behaviours within individuals. We also predicted that due to physiological costs, high salinity should decrease the overall investment in reproductive behaviours and that this effect may differ for aggression and courtship.

## Materials and Methods

### Fish origin and maintenance

The study was conducted in the School of Biological Sciences, Monash University (Australia), between October 2009 and April 2010. The first generation laboratory-born desert gobies used in the experiment were descended from a breeding colony of individuals that were originally collected as juveniles from waterholes and springs located within the Neales River system in the Lake Eyre Basin of South Australia^37^. Within their natural habitat, desert gobies have been observed inhabiting a wide range of salinities (<5 ppt to ~100 ppt; personal observations 2007–2012) that can also fluctuate, either gradually or suddenly (i.e. within a matter of hours or days), depending on habitat type, rate of evaporation, rainfall, and hydrology^39^. At the two main sites where desert gobies were collected to establish our breeding population, water samples taken at four separate time points (per site) revealed salinities ranging from 1.3–80 ppt (i.e. Ockenden Springs: 2.7–12 ppt; Peake Creek: 1.3–80 ppt).

Fish were separated by sex at maturation and housed in 80–250 l aquaria that had a sand substrate with clay pots and plastic structures as shelter. The aquaria were maintained at a temperature of 23–26 °C and a salinity of ~6 ppt, with a 12:12 h light:dark cycle. The fish were fed 1–2 times a day, alternating between commercial fish food pellets and frozen *Artemia* brine shrimp.

The procedures detailed in this study were approved by the Biological Sciences Animal Ethics Committee of Monash University, Australia (permit no. BSCI/2007/12), and comply with all the relevant Federal and State laws of Australia.

### Experimental setting

**Figure 1:**
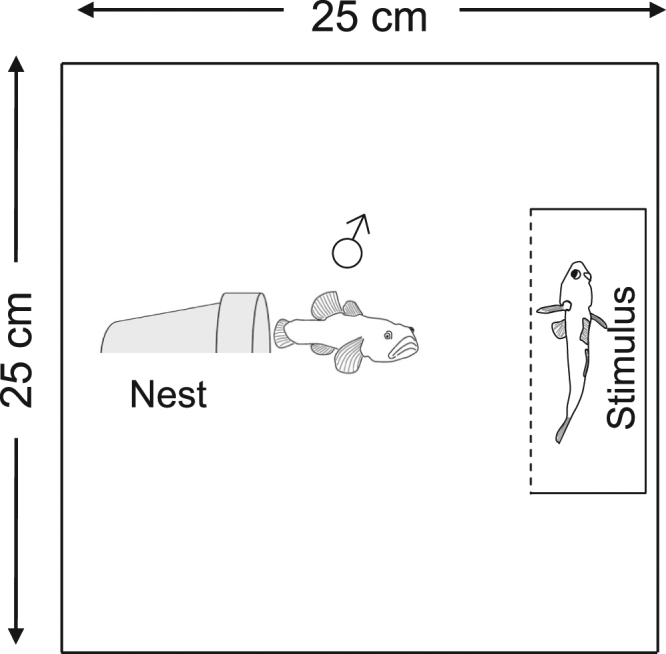
Schematic top-view of the experimental aquaria. Fish images (lateral-view) by Nicholas Deal, reproduced with permission.

Before being used in the experiment, each fish was weighed to the nearest 0.01 g in a container of water on an electronic balance. Focal males were also photographed in a container of water that had 1 cm marks on the bottom, which allowed the total lengths of the males to be later assessed using ImageJ 1.51k software (National Institute of Health, USA). At the beginning of a trial, a male desert goby was introduced into an experimental arena of 25 cm × 25 cm × 20 cm (length × width × water depth) with a 3 cm layer of sand on the bottom and a halved clay flowerpot (diameter: 6.5 cm, length: 6.5 cm) as a nesting resource. The entrance of the nesting resource faced the stimulus fish compartment (see ‘Behavioural assessments’ below), which consisted of a transparent container, 5 cm × 15 cm × 22 cm (length in the direction of nest entrance × width × water depth), placed inside the experimental arena, directly against one of its walls (Fig. 1).

Due to a limited number of tanks available for the study, we ran replicates in multiple batches with approximately the same number of replicates of each treatment (see below) in each batch. Physiological demands of the environment were manipulated using two salinity treatments, low (5 ppt) and high (35 ppt), with the latter representing a higher metabolic cost than the former^35^. Fish in the laboratory have been observed to engage in reproductive behaviours at both salinity levels (while eggs do not seem to develop normally in salinities approaching 50 ppt). Hence, the salinities used in our study fell within the range at which reproduction is expected to occur. We applied the salinity acclimatisation schedule of Lehtonen *et al*.^38^. Briefly, after the focal male had been introduced into the experimental arena, we gradually (over 24 hours) increased salinity from the initial salinity of ~6 ppt to an intermediate salinity of ~19 ppt. We then gave the male several days (n = 90 measurements, mean ± SE: 4.8 ± 0.2 days) to acclimate to these conditions. Salinity was then either gradually (over 24 hours) reduced to ~5 ppt in low salinity replicates (n = 44, focal male mean body mass ±SE: 2.89 ± 0.15 g, total length: 5.88 ± 0.10 cm) or further increased to ~35 ppt in high salinity replicates (n = 46, focal male mean body mass ± SE: 2.74 ± 0.12 g, total length: 5.79 ± 0.09 cm). The acclimatisation schedule was applied so that focal males in both low and high salinity replicates were subjected to substantial changes in salinity. Each focal male was exposed to only one salinity level, and was randomly assigned to its salinity treatment. After reaching the target salinity, the focal males were allowed to acclimate for a week.

### Behavioural assays

The behaviours of low and high salinity focal males were individually measured in two successive trials. The first trial was initiated by adding a stimulus individual, either a mature female or male, into the stimulus compartment that was positioned in front of the focal male’s nest entrance. After an acclimation period of 3.5 minutes, the behaviours of the focal male were sampled at 24 observation periods, each lasting 25 seconds, that were evenly distributed throughout the 96 minute trial. Hence, the total time of observation was 10 minutes per trial. The procedure for collecting data on behaviour of male gobies followed previously published procedures^38,40,41^. Briefly, to prevent disturbance to the fish, the observer was seated in the dark, away from the tanks, which were lit from above with fluorescent lamps. The focal male was recorded as interacting with the stimulus individual when it was within 4 cm of the stimulus compartment, with its body oriented towards the stimulus individual while engaged either in aggression displays (in the case of the male stimuli, these included fin displays, attacks and biting attempts^38,42^) or courtship behaviour (in the case of the female stimuli, these were fin displays, ‘hopping’ displays, and ‘leading swims’ towards the nest^40,41^). Both the ‘total time’ engaged in aggression/courtship and the number of ‘bouts’ of aggression/courtship are relevant and widely used measures of sexual behaviour in fish^43^. For the analyses, we quantified behaviour of the focal males as the overall time, out of the total of 600 seconds observed per trial, that the male performed aggression/courtship. The number of distinct bouts of aggression/courtship during the observed time was also counted and the results for this variable are provided in the Supplementary materials. These two measures were highly correlated (Pearson’s correlation, r = 0.96, df = 178, p < 0.001) and give very similar results. However, we point out instances when the results provided by the two variables differ qualitatively.

Approximately twenty minutes after completion of the first trial, each male was subjected to a second trial using identical protocols as in the first. A different stimulus individual was used for the two trials. The data points of the second (but not first) trial have been used in an earlier study that assessed the effects of social exposure and salinity on behavioural traits^38^.

Focal males in each salinity treatment were either tested in the presence of (1) different stimulus males in trials 1 and 2 to assess consistency of male aggression (n_low salinity_ = 11, n_high salinity_ = 12), (2) different stimulus females in trials 1 and 2 to assess consistency of male courtship (n_low_ = 11, n_high_ = 11), or (3) one stimulus male and one stimulus female to assess the relationship between male aggression and courtship (n_low_ = 22, n_high_ = 23). To account for order effects in (3), the presentation order of male and female stimuli was alternated between trials. In other words, in approximately a half of the replicates, male fish was presented first (n_low_ = 11, n_high_ = 12), whereas female fish was presented first in the remaining replicates. Fish were never used more than once as a focal individual (with some of the focal males being also used once as a stimulus). We only used sexually mature males and females, which was determined by their distinct nuptial colouration and pronounced distended bellies, respectively.

### Behavioural consistency within and between salinities

Consistency of behaviour (aggression or courtship) within each salinity treatment was assessed as Pearson’s correlations between the first and second trial of measurement of the same behaviour (aggression or courtship). To achieve normality, the data were square root transformed for these and the subsequent analyses. The difference in strength of the behavioural correlations from low and high salinity were compared using two-tailed testing (based on Fisher’s z) in the ‘cocor’ statistical package (1.1–3), available online at: http://comparingcorrelations.org.

### Relationship between aggression and courtship relative to salinity

The relationship between aggression and courtship was assessed for males that were subject to presentations of both a stimulus male and stimulus female. The level of consistency in the low versus high salinity treatments was assessed by comparing the two correlation values from low and high salinity (as above).

### Effect of salinity treatments on the expression levels of aggression and courtship

We also assessed the effect of salinity on the overall rate of aggression and courtship. We did this over the entire data-set by applying a linear mixed effects model. Salinity treatment and the sex of the stimulus fish were added as fixed factors and body mass was added as a covariate. The latter was included because desert goby aggression may be influenced by male size^42^, while studies on other goby species have suggested that small and large males adjust their reproductive behaviours differently in response to different environmental factors^34,44,45^. Because the data included two trials of behavioural assessment performed by the same focal male (i.e. non-independence), focal male ID was included as a random factor. If the interaction between salinity and body mass was found to be non-significant (p > 0.10), we then refitted the model without the interaction (as per Crawley^46^) before interpreting the main effects.

### Data availability

Data are available in Supplementary Table S1.

## Results

### Behavioural consistency within and between salinities

**Figure 2:**
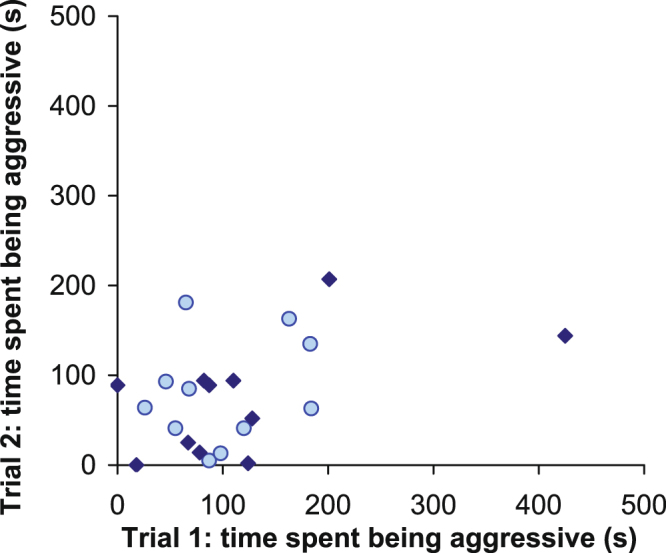
Time spent being aggressive by focal males in trials 1 and 2. The circles and diamonds indicate the low (n = 11) and high (n = 11) salinity treatments, respectively.

The time male spent being aggressive was not significantly correlated between the first and second trial in low (r_Pearson_ = 0.16, df = 9, p = 0.64) or high (r_Pearson_ = 0.43, df = 9, p = 0.19) salinity treatments. The effect of salinity treatment on the level of consistency of aggression was not significant (comparison between correlations in the two salinity treatments based on Fisher’s z, z = 0.5971, p = 0.55). Even with the two salinity levels combined, aggression level did not significantly correlate between the two trials (r_Pearson_ = 0.34, df = 20, p = 0.12; Fig. 2).

**Figure 3:**
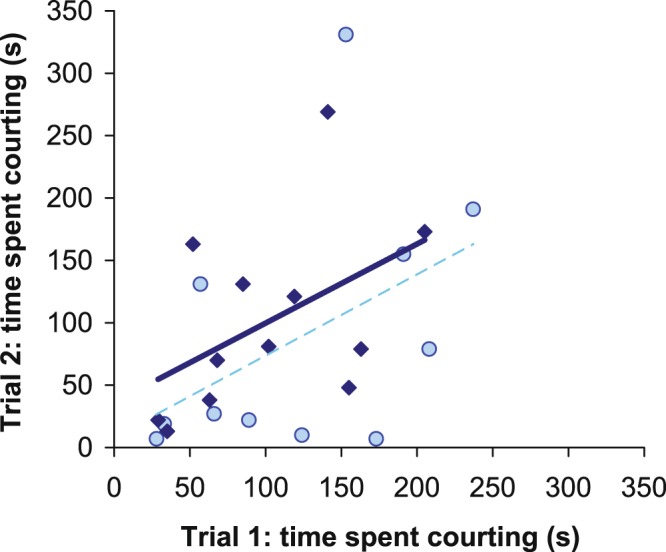
Time spent courting females by focal males in trials 1 and 2. The circles and diamonds indicate the low (n = 11, dashed line) and high (n = 12, solid line) salinity treatments, respectively.

The time the focal male spent courting was not significantly correlated between the first and second trial in the low salinity treatment (r_Pearson_ = 0.50, df = 9, p = 0.12). However, when courtship level was measured as the number of courtship bouts, this correlation was significant (Supplementary materials). In the high salinity treatment, there was a marginally non-significant tendency for the time spent courting (r_Pearson_ = 0.56, df = 10, p = 0.057) to correlate between the two trials. The effect of salinity on the consistency of courtship was not significant (comparison between correlations in the two salinity treatments, Fisher’s z, z = 0.1884, p = 0.85). With the two salinity levels combined, courtship was consistent, as indicated by the significant correlation between the two trials (r_Pearson_ = 0.49, df = 21, p = 0.019; Fig. 3).

### Relationship between aggression and courtship relative to salinity

**Figure 4:**
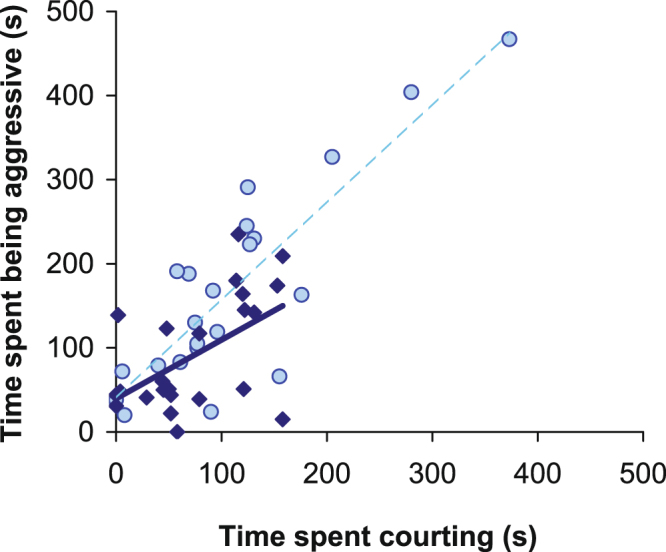
Correlation between time being aggressive and time spent courting (n = 23), as performed by the same focal males. The circles (with dashed line) and diamonds (solid line) indicate the low and high salinity treatments, respectively.

In the group of males that were tested in the presence of one stimulus male and one stimulus female, the time males spent being aggressive was highly correlated with the time spent courting in the low salinity treatment (r_Pearson_ = 0.66, df = 20, p < 0.001). In the high salinity treatment, this pattern was similar, albeit weaker and marginally non-significant when measured as the time spent engaged in the two behaviours (r_Pearson_ = 0.38, df = 21, p = 0.070), but significant when measured as the number of bouts of behaviour (Supplementary materials). Overall, salinity treatment did not have a significant effect on the strength of the correlation between aggression and courtship (Comparison of the two correlations based on Fisher’s *z*, z = 1.235, p = 0.22). The correlation between aggression and courtship over the two salinity levels was highly significant (r_Pearson_ = 0.58, df = 43, p < 0.001; Fig. 4).

### Effect of salinity treatments on the expression levels of aggression and courtship

**Figure 5:**
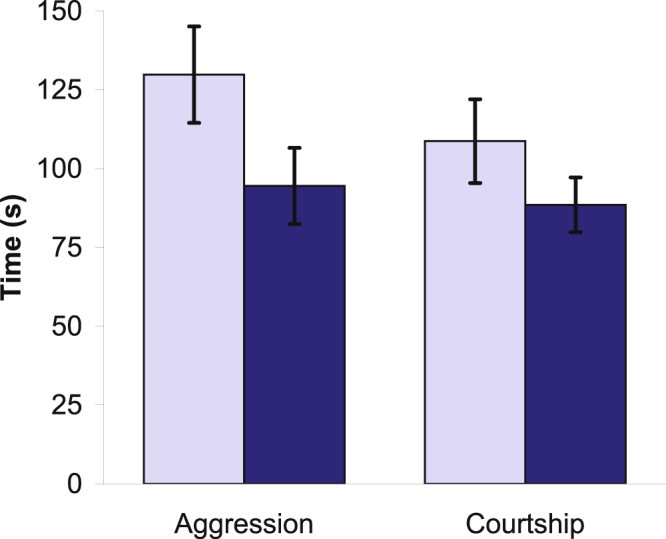
The overall time spent being aggressive and courting in low (light blue columns, n_aggression_ = 44, n_courtship_ = 44) and high salinity (dark blue columns, n_aggression_ = 45, n_courtship_ = 47) conditions.

The interaction between salinity treatment × behavioural type was not significant (χ^2^ = 1.549, df = 1, p = 0.21). The refitted model with only main effects indicated that male body mass did not have a significant effect on the behaviours (linear mixed model, t_87_ = 0.3140, p = 0.75). The effect of treatment, in turn, was marginally not significant (linear mixed model, t_87_ = 1.707, p = 0.091): focal males tended to use more time for the measured behaviours in low than high salinity treatment (Fig. 5). The latter pattern was non-significant when the numbers of bouts of behaviours were considered (Supplementary materials). Finally, the sex of the stimulus individual had a significant effect (linear mixed model, t_89_ = 2.177, p = 0.032): males spent more time being aggressive than courting (Fig. 5).

## Discussion

We found that courtship behaviour was moderately consistent over the two trials with different stimulus individuals, whereas aggression did not show a significant consistency. Nevertheless, the two behaviours were significantly correlated: males that were aggressive also courted more. Salinity treatment, in turn, did not affect the consistency of the two behaviours, or the association between them, although overall males tended to use more of their time in these behaviours in the low salinity treatment.

Our results indicate a significant consistency in courtship displays, but a non-significant consistency in aggression. These findings are similar to those reported in field crickets, *Gryllus veletis*, where male signalling effort was found to be highly repeatable, but aggressive behaviour was not^17^. It is worth noting, however, that in our study, different stimulus individuals were used in each trial. This is relevant because if focal males were adjusting their aggression or courtship due to the appearance or behaviour of the stimulus individuals (as suggested to be the case in a number of other fish species^47–50^, including desert gobies^41^), such behavioural adjustments could have deflated the correlation estimates. Such a possibility is also consistent with a higher estimate for the consistency of courtship behaviour being found when using the same stimulus individual for the two successive rounds of assessment^41^. The seemingly higher consistency of courtship compared to that of aggression in the current study may be due to differences between stimulus males being more important for focal male responses than differences between females. In particular, different females may elicit fairly similar levels of interest from focal males because attracting any female to add eggs to an empty nest will improve the male’s reproductive success. In contrast, intruding males may, depending on their behaviour or physical attributes, pose quite different levels of threat, hence inducing more varied responses in the focal male. We cannot completely rule out the possibility that males were more fatigued from the displays of aggression than courtship, which could have resulted in a lower consistency of aggression. However, based on our observations, courtship in desert gobies seems to be at least as taxing as aggressive displays, and males in the current study indeed invested less time in courtship than aggression. Furthermore, the rest of our results did not support the idea that fatigue caused the decrease in behavioural consistency.

Indeed, despite potential effects of fatigue on focal male behaviour, as well as potential effects from differences between stimulus fish, we found a strong correlation between the rates of aggression and courtship. Furthermore, this correlation remained high under both environmental contexts we tested: the salinity treatment did not significantly affect the consistency. These findings are important because suites of correlated behaviours across environmental contexts are often considered as behavioural syndromes or personalities^51^. The presence of such co-existing behavioural types in populations has important ecological and evolutionary implications, as they can influence reproductive success, competitive interactions, dispersal, and responses to environmental variation^52–54^, all of which are relevant for the life-history of desert gobies^37,42^. In this regard, the positive correlation between aggression and courtship could be due to ‘bold’ individuals being more aggressive, as well as being more prone to courtship, when females approach their nest^51,55^. In species that have very different aggression and sexual display behaviours, the two behaviours may be expected to be less tightly associated than when they have many shared behavioural elements, as is the case in desert gobies (both aggression and courtship involve intensified colours, erect fins and jerky body movements).

Not only was consistency of aggression and courtship similar in the two salinity treatments but the correlation between aggression and courtship was also unaffected by salinity. We found only a non-significant tendency for males to show lower levels of the two behaviours in the high salinity treatment. An earlier study assessing the levels of aggression and courtship in male desert gobies, using a smaller data-set, found that while the influence of prior social experience on aggression differed between salinity exposures, there was no significant overall salinity effect on the levels of aggression or courtship^38^. Together, these results indicate that despite the physiological costs associated with adjustments to higher salinity levels^35^, species such as the desert goby can retain the ability and motivation to perform these key reproductive behaviours under a wide range of salinity conditions. In contrast, species living in more benign or stable environments are probably not able to perform over such wide range of environmental conditions. For example, guppies, *Poecilia reticulata*, had limited local adaptation to harsh environmental conditions, which was explained with the costs of adapting to stressful environments^20^. In mosquitofish, *Gambusia affinis*, individuals from families with no historical exposure to salinity had lower survival levels at elevated salinity levels than descendents of individuals from brackish marshes^56^.

Other factors may also have contributed to our results. First, we note the possibility that salinity might have had effects that were too small to be detected with the sample sizes used in this study. However, we consider it unlikely that a larger data-set would have revealed the predicted drop in behavioural consistency under high salinity. This is because, if anything, the correlation between successive aggression measurements was numerically larger in high (r_Pearson_ = 0.43) compared to low (r_Pearson_ = 0.16) salinity, i.e. a pattern that was opposite to the one we had predicted. Second, high levels of within individual variation, as manifested in our study by the low-to-moderate correlations, could potentially mask any salinity effects on behavioural consistency. It is worth noting, however, that even in the analysis involving the entire data-set, we found only limited evidence for effects of salinity on the combined intra- and intersexual behaviours. Finally, energetic costs may have been higher when the salinity level was changing (i.e. during the experiment’s acclimation period) rather than when the fish had settled into a consistent salinity level (i.e. when their behaviours were measured). In other words, it is possible that salinity effects would have been stronger either during fluctuations in salinity levels or after very long (chronic) exposure times. Therefore, future work assessing the effects of salinity exposures of varying lengths would be highly interesting.

The males spent, on average, more time being aggressive towards a stimulus male than they spent courting a female. This result warrants future investigations, especially because we currently do not know whether the energetic costs of these two behaviours are the same per unit time. For example, if courtship is physiologically more taxing than attacks and aggressive displays (without a physical contact), the investment into the two behaviours might actually have been roughly equal. Interestingly, the size of the males did not affect the time they spent on aggression or courtship. This result may appear to contrast with those reported in an earlier study, which showed that smaller desert goby males were more aggressive^42^. The difference in results between the studies could be due to a smaller body size of the largest males, or smaller mean body size, in the current study.

To conclude, we found that aggressive individuals also performed more intense courtship displays. This positive relationship was not significantly affected by salinity, suggesting that the behavioural correlation is robust over different environmental contexts. This can be seen as evidence for different personality types in desert gobies. The absolute levels of aggression and courtship displays were not markedly affected by our salinity treatments, although overall, males had a tendency to have slightly reduced levels of the two types of reproductive behaviours in the high salinity treatment. Together, these results suggest that in species that are commonly exposed to varying salinity levels, such as the desert goby, investment into current reproductive behaviours can occur at typical or near-to-typical levels under a range of different salinity conditions.

## Electronic supplementary material

Supplementary materials
