# Supplementary material for: Aggressive desert goby males also court more, independent of the physiological demands of salinity
Source: Sci Rep. 2018 Jun 19;8:9352. doi: 10.1038/s41598-018-27651-3 (PMC6008469; doi:10.1038/s41598-018-27651-3)
Supplement: Supplementary file 1 — Supplementary materials [file 41598_2018_27651_MOESM1_ESM.pdf]

Aggressive desert goby males also court more, independent of the physiological demands of salinity

Topi K. Lehtonen, P. Andreas Svensson and Bob B. M. Wong

Supplementary material: Results with respect to bouts of behaviours

Behavioural consistency within and between salinities

The number of aggression bouts was not significantly correlated between the first and second trials in low ($r_{Pearson}$ = 0.23, df = 9, p = 0.51) or high ($r_{Pearson}$ = 0.41, df = 9, p = 0.21) salinity. The level of consistency of aggression was not significantly different under the two salinity levels (comparison between correlations in the two salinity treatments, based on Fisher's z: z = 0.4104, p = 0.68). The number of aggression bouts was not consistent between the two trials even when the two salinity levels were combined ($r_{Pearson}$ = 0.31, df = 20, p = 0.16).

The number of courtship bouts by the focal male was significantly correlated between the first and second trial in low salinity ($r_{Pearson}$ = 0.63, df = 9, p = 0.039), and there was a marginally non-significant tendency for the courtship bouts to be correlated between the two trials in high salinity ($r_{Pearson}$ = 0.56, df = 10, p = 0.058). The effect of salinity on the consistency of courtship bouts was not significant (comparison between correlations in the two salinity treatments, Fisher's z: z = 0.2089, p = 0.83). We therefore combined the two salinity levels and found that in this combined dataset, courtship was consistent over the two trials ($r_{Pearson}$ = 0.56, df = 21, p = 0.005).

Relationship between aggression and courtship relative to salinity

In the group of males that were tested in the presence of one stimulus male and one stimulus female, the number of bouts of aggression was highly correlated with the number of courting bouts, both in low ($r_{Pearson}$ = 0.60, df = 20, p = 0.003) and high ($r_{Pearson}$ = 0.46, df = 21, p = 0.028) salinity. Overall, salinity did not have a significant effect on the strength of the correlation between aggression and courtship (Comparison of the two correlations base on Fisher's z: z = 0.6390, p = 0.52).

The correlation between aggression and courtship bouts over the two salinity levels was highly significant ($r_{Pearson} = 0.55$, df = 43, $p < 0.001$).

Effect of salinity on the expression levels of aggression and courtship

With regards to bouts of behaviour, we found that there was no significant salinity × behavioural type interaction ($\chi^2 = 1.483$, df = 1, p = 0.22). The refitted model with the main effects indicated that the effects of male body mass (linear mixed model, $t_{87} = 0.4473$, p = 0.66) and salinity (linear mixed model, $t_{87} = 1.443$, p = 0.16) were also non-significant. Finally, stimulus individual sex had a significant effect (linear mixed model, $t_{89} = 2.389$, p = 0.019): males exhibited a higher number of aggression than courtship bouts.

Supplementary material: Raw data supporting the results

Table S1. Time spent being aggressive towards a stimulus male, or courting a stimulus female. The numbers of bouts of these behaviours in the two rounds of trials by each of the 90 focal males, salinity treatment information, focal male body sizes, and stimulus fish sex are also given.

**Male ID**	**Body mass**	**Total length**	**Salinity**	**Stimulus1 sex**	**Stimulus2 sex**	**Time1**	**Bouts1**	**Time2**	**Bouts2**
PC454	2.99	60.39	low	female	female	191	23	155	18
OSF1T6	2.51	56.2	low	female	female	28	2	7	1
PCT2	4.12	64.38	low	female	female	173	31	7	3
3DMPC5	2.22	53.04	low	female	female	153	39	331	67
6F1OS1	2.18	53.3	low	female	female	33	4	19	1
6DMOS2	1.83	50.27	low	female	female	237	29	191	25
10ANR1	4.32	67.24	low	female	female	57	9	131	24
PCQ22C	2.35	55.04	low	female	female	66	10	27	4
PCQ25B	2.16	51.53	low	female	female	124	18	10	2
PCQ23A	2.42	57.02	low	female	female	208	37	79	16
Q26C7	2.24	55.1	low	female	female	89	15	22	4
PC458	4.77	67.14	low	female	male	90	9	24	3
OSF1T3	2.44	56.11	low	female	male	61	9	83	14
OSF1T5	2.57	57.21	low	female	male	75	11	130	15
NRT4	5.25	72.95	low	female	male	131	16	230	25
3DMPC4	2.71	69.05	low	female	male	69	10	188	42
PCT6	2.11	53.44	low	female	male	280	41	404	54

6DMOS1	1.99	52.16	low	female	male	124	16	245	42
10AOS1	2.17	55.22	low	female	male	176	20	163	26
10BPC3	1.99	52.27	low	female	male	77	15	100	18
PCQ22D	2.68	58.51	low	female	male	127	20	223	35
Q26C15	2.1	54.57	low	female	male	40	5	79	9
PC457	4.18	67.71	low	male	female	291	37	125	15
II418NRF1	5.54	74.66	low	male	female	38	6	0	0
OSF1T2	4.87	70.19	low	male	female	327	58	205	33
NRT3	3.45	64.85	low	male	female	72	11	6	2
3DMPC7	3.61	61.22	low	male	female	467	75	373	51
PCT7	2.04	52.21	low	male	female	66	9	155	27
6DMPC1	1.88	51.8	low	male	female	191	28	58	8
10APC1	3.45	60.08	low	male	female	20	3	8	1
10BMIX2	2.88	60.2	low	male	female	105	18	77	13
PCR11c	1.95	55.7	low	male	female	168	32	92	18
R11C13	2.14	56.17	low	male	female	119	20	96	18
PC426	4.24	67.34	low	male	male	120	21	41	5
PC418	4.11	68.46	low	male	male	55	6	41	6
PCT3	3.4	63.87	low	male	male	26	7	64	14
3PCE	2.96	60.14	low	male	male	65	14	181	43
6F1NR1	2.3	53.37	low	male	male	87	9	5	0
OMIX2	1.83	52.19	low	male	male	46	4	93	12
10AMIX4	2.98	60.7	low	male	male	163	21	163	30
10BMIX3	2.96	58.55	low	male	male	184	39	63	12
PC11A	2.12	50.49	low	male	male	68	11	85	17
PCX	1.99	54.09	low	male	male	183	33	135	28
Q22C6	2.01	53.07	low	male	male	98	21	13	2

PC425	2.22	55.56	high	female	female	29	4	22	4
PC415	3.4	64.03	high	female	female	163	11	79	6
PCT1	3.94	66.19	high	female	female	68	14	70	14
3DMPC6	3.42	62.64	high	female	female	85	12	131	18
6F1OS2	3.16	60	high	female	female	63	9	38	5
10AMIX2	2.59	55.84	high	female	female	205	29	173	36
10ANR2	2.2	50.08	high	female	female	119	16	121	16
10BPC1	2.73	56.09	high	female	female	155	25	48	6
NR11A	2.29	55.39	high	female	female	52	7	163	25
PCR11B	2.11	56.76	high	female	female	102	16	81	14
Q31C2	1.83	50.96	high	female	female	35	8	13	2
Q31C11	2.44	58.29	high	female	female	141	22	269	47
PC417	3.86	66.26	high	female	male	44	7	60	7
NRT2	3.54	62.77	high	female	male	52	8	44	6
NRT5	4.13	65.06	high	female	male	0	0	44	6
3DMPC2	4.35	69.08	high	female	male	114	21	180	36
PCT4	4.18	68.39	high	female	male	48	13	123	31
6DMPC2	1.79	49.21	high	female	male	158	28	15	4
10AMIX1	2.1	53.07	high	female	male	52	10	22	5
10BPC2	1.7	48.51	high	female	male	79	19	39	7
PCR11A	1.95	54.73	high	female	male	153	27	174	40
Q25C12	2.5	59.81	high	female	male	0	0	31	5
R12C10	2.5	58.37	high	female	male	50	6	51	8
PC416	2.93	60.07	high	male	female	142	30	131	22
II419NRF1	3.6	66.49	high	male	female	139	25	2	1
MIXF1T1	1.64	48.43	high	male	female	51	4	121	13
NRT1	3.73	61.5	high	male	female	164	29	120	12

3DMPC1	1.79	53.61	high	male	female	235	46	116	21
PCT5	1.93	51.25	high	male	female	117	13	79	13
OMIX1	2.21	53.77	high	male	female	50	5	45	8
10BNR1	2.25	55.67	high	male	female	209	40	158	32
PCQ25A	2.48	55.47	high	male	female	48	10	4	1
PCQ25C	2.18	56.82	high	male	female	0	0	58	9
Q31C3	2.35	57.96	high	male	female	145	30	122	21
R12C9	2.9	60.58	high	male	female	41	6	29	6
PC452	3.47	62.87	high	male	male	425	73	144	22
II415NRF1	3.08	60.05	high	male	male	82	10	94	8
PC413	4.85	71.65	high	male	male	87	13	89	18
3DMPC3	3.84	64.99	high	male	male	0	0	89	13
6F1OS3	2.09	53.57	high	male	male	67	8	25	4
6DMOS3	2.36	53.29	high	male	male	124	24	2	1
10AMIX3	2.37	54.71	high	male	male	110	19	94	12
PC11B	2.28	53.17	high	male	male	201	29	207	28
PCQ22B	2.01	53.55	high	male	male	128	24	52	8
PCQ25D	2.45	55.18	high	male	male	78	14	14	2
MIXY	2.24	52.59	high	male	male	18	3	0	0
